# Supplementary material for: MetaRibo-Seq measures translation in microbiomes
Source: Nat Commun. 2020 Jun 29;11:3268. doi: 10.1038/s41467-020-17081-z (PMC7324362; doi:10.1038/s41467-020-17081-z)
Supplement: Supplementary file 10 — Supplementary Data 7 [file 41467_2020_17081_MOESM10_ESM.zip › File2/Confidence_High_Taxonomy/438093_out.krona.html]

Javascript must be enabled to view this page.

members
magnitude
magnitudeUnassigned
count
unassigned
taxon
rank

438093\_out

15

2
superkingdom
15

15
phylum
1224

1236
class
15

91347
order
15


SRS019068\_contig\_number\_9106SRS019068\_contig\_number\_50891SRS046717\_contig\_number\_contig-100\_930.931SRS971427\_contig\_number\_31263
543
family
4
15

1
genus
547

1
species
539813

SRS1055050\_contig\_number\_contig-100\_645.646

genus
561
8

8
species
562

SRS012273\_contig\_number\_40063SRS017191\_contig\_number\_15975SRS017521\_contig\_number\_31412SRS049402\_contig\_number\_11220SRS076876\_contig\_number\_contig-100\_592.593SRS144362\_contig\_number\_38278SRS146888\_contig\_number\_13604SRS147022\_contig\_number\_14108

570

SRS050422\_contig\_number\_14543
2
1
genus

1

SRS143070\_contig\_number\_9998
573
species
